# Contemporary Mouse Models in Glioma Research

**DOI:** 10.3390/cells10030712

**Published:** 2021-03-23

**Authors:** William H. Hicks, Cylaina E. Bird, Jeffrey I. Traylor, Diana D. Shi, Tarek Y. El Ahmadieh, Timothy E. Richardson, Samuel K. McBrayer, Kalil G. Abdullah

**Affiliations:** 1 Department of Neurological Surgery, University of Texas Southwestern Medical Center, Dallas, TX 75235, USA; william.hicks@UTSouthwestern.edu (W.H.H.); cylaina.bird@utsouthwestern.edu (C.E.B.); jeffrey.traylor@utsouthwestern.edu (J.I.T.); tarek.elahmadieh@phhs.org (T.Y.E.A.); 2 Department of Radiation Oncology, Brigham and Women’s Hospital and Dana-Farber Cancer Institute, Harvard Medical School, Boston, MA 02215, USA; dshi1@partners.org; 3 Department of Pathology, Glenn Biggs Institute for Alzheimer’s and Neurodegenerative Diseases, University of Texas Health San Antonio, San Antonio, TX 75229, USA; richardson.te@gmail.com; 4 Children’s Medical Center Research Institute, University of Texas Southwestern Medical Center, Dallas, TX 75390, USA; 5 Harrold C. Simmons Comprehensive Cancer Center, University of Texas Southwestern Medical Center, Dallas, TX 75235, USA; 6 Peter O’Donnell Jr. Brain Institute, University of Texas Southwestern Medical Center, Dallas, TX 75235, USA

**Keywords:** glioma, genetically engineered mouse models (GEMM), isocitrate dehydrogenase (IDH), patient-derived xenograft (PDX), mouse model

## Abstract

Despite advances in understanding of the molecular pathogenesis of glioma, outcomes remain dismal. Developing successful treatments for glioma requires faithful in vivo disease modeling and rigorous preclinical testing. Murine models, including xenograft, syngeneic, and genetically engineered models, are used to study glioma-genesis, identify methods of tumor progression, and test novel treatment strategies. Since the discovery of highly recurrent isocitrate dehydrogenase (IDH) mutations in lower-grade gliomas, there is increasing emphasis on effective modeling of IDH mutant brain tumors. Improvements in preclinical models that capture the phenotypic and molecular heterogeneity of gliomas are critical for the development of effective new therapies. Herein, we explore the current status, advancements, and challenges with contemporary murine glioma models.

## 1. Introduction

Diffuse gliomas are the most common primary tumor of the central nervous system (CNS) and are currently classified as lower-grade glioma (WHO grade II and III) or glioblastoma (GBM; WHO grade IV) based on a combination of histologic and molecular features [1]. Based on the histologic similarity of the tumor to glial cells, diffuse glioma is a broad term encompassing astrocytoma, oligodendroglioma, each of their anaplastic variants, and GBM [1]. Lower-grade gliomas tend to be slower growing and are less aggressive than higher grade gliomas, with the diagnosis of GBM conferring a dismal prognosis [1]. Despite advances in treatment, patients with GBMs have a median survival of 15 months and a 5-year survival rate of <10% with maximal resection and concomitant chemotherapy and radiation [1]. The intractability of these tumors highlights the need for clinical testing of new therapies that display robust activity in accurate mouse models of glioma.

In vivo cancer modeling provides numerous advantages over in vitro modeling. Over 80% of the genes in the mouse genome have direct human orthologs, thereby leading to adoption of the mouse as the dominant model organism for cancer biology and cancer therapy studies [2]. Recent advances in genetic engineering have enabled the production of mouse models of glioma that increasingly mimic the microenvironmental and genomic characteristics of human brain tumors. The genetic landscape of glioma is characterized by alterations in genes encoding epidermal growth factor receptor (EGFR), phosphate and tensin homolog deleted on chromosome 10 (PTEN), neurofibromatosis 1 (NF1), RAS, TP53, and cyclin dependent kinase inhibitor 2 (CDKN2A/B), among others, leading to cell proliferation and tumorigenesis [3,4]. Recently, mutations in isocitrate dehydrogenase 1 and 2 (IDH1/2) have been identified in the majority of lower-grade gliomas and a relatively small subset of GBMs [3,5]. As lower-grade gliomas invariably progress to secondary GBMs, evaluating the role of IDH directed therapy is important for patient care.

Recapitulation of the diffuse and infiltrative nature of glioma has been challenging to achieve in murine glioma models. Gliomas do not display the well-circumscribed morphology typical of many other solid tumors. Therefore, representing this unique property of these cancers in mice is desirable in order to accurately model tumor-stroma interactions and glioma cell behavior. Over the last 10 years, substantial advances in the understanding of the molecular pathogenesis of glioma have prompted updates to the WHO classifications system for glioma (combining both histopathological and molecular tumor characteristics) and have guided efforts to develop new targeted therapies and murine models for this disease [5]. The genetic diversity, inter- and intra-tumoral heterogeneity, and extensive interaction with brain parenchyma displayed by gliomas lead to late clinical detection, resistance to treatment, and universal tumor recurrence following therapy. These features highlight the need for efficient and representative preclinical mouse models of glioma [6,7]. In this review, the evolution, history, and current status of contemporary glioma mouse models is discussed.

## 2. Evolution of Cancer Mouse Models

**Figure 1:**
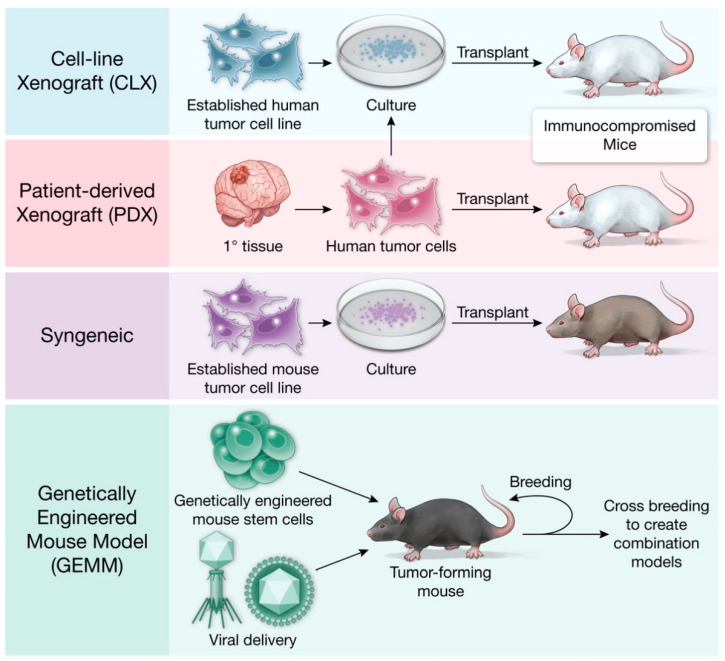
Murine preclinical cancer modeling.

Cancer mouse models evolved alongside advances in molecular and medical technology and vary in cost and immune status (Table 1). A visual summary of the distinct types of mouse models discussed in this article are provided in Figure 1.

The first cancer animal model was the xenograft model. Historically, this model achieved tumor growth through hetero-transplantation of human cancer cells into immune-privileged sites like the guinea pig eye or hamster cheek-pouch [8]. While effective as an animal culture of the tumor, these early models provided limited opportunity for study of tumor interaction with native tissue cell types and precluded orthotopic transplantation (into the organ of origin). This challenge was overcome in 1953, when cortisone-treated, irradiated rat xenograft models grew transplanted human tumors [9]. Rygaard and Paulson (1969) further established an immune-compromised host as a critical tool for effective tumor xenograft transplantation [10]. Engraftment rates were significantly improved by immune-compromised mice which led to widespread adoption of xenograft models in the cancer research field [11].

Around the time that the first xenograft models were established, syngeneic models were created to facilitate the identification of effective chemotherapies [12]. From the 1950s to 1970s, the National Cancer Institute conducted chemotherapeutic screening programs using syngeneic models of sarcoma 180, L1210 leukemia, B16 melanoma, and P388 leukemia, among others [12]. Syngeneic models are created through the use of carcinogens or genetic modification to induce tumorigenesis or by leveraging spontaneous tumor formation in the mouse [13,14]. Malignant transformation can be induced in vitro or in vivo. If primary cells are transformed in vitro, they can be introduced to an organism of the same species. C57BL/6, BALBc, and FVB/N are common mouse species used in syngeneic models and have been critical for the preclinical evaluation of experimental therapeutics [13,14]. Xenograft and syngeneic modeling approaches have been applied extensively to glioma research, as summarized below.

Genetically engineered mouse models (GEMMs) were first established when oncogenic viral DNA was detected in the adult mouse following transfection of the mouse embryo with simian virus 40 (SV40) [15]. In the 1980s, there was a rapid expansion of transgenic GEMMs with the creation of onco-mice [16,17,18]. These onco-mice have tumorigenic DNA, often known or proposed oncogenes, introduced into their genome to create a mouse predisposed to tumor formation. Subsequent models placed the oncogene under tissue specific promoters, like the pairing of the immunoglobulin enhancer to the Myc gene to model B-cell lymphomas or the hormone inducible mouse mammary tumor virus-Ras mouse to model breast cancer [17,19]. Gu et al. (1993) established the Cre-loxP system as a conditional gene targeted tool for genetic recombination [20]. A similar model was simultaneously developed utilizing the Flp-FRT system [21]. These molecular tools led to the development of conditional inducible mouse models of cancer and are discussed at greater length later in the review. As our genetic and molecular understanding of specific cancers, including glioma, continues to grow, individual genetically engineered mice can be bred to generate combinatorial genetic defects that better resemble the multi-allelic abnormalities in human cancer.

## 3. Xenograft Models

Historically, orthotopic high grade glioma (HGG) xenograft models were created with patient-derived cell lines or established cell lines. In cell-line xenograft models (CLX), cells are implanted into the desired location in the mouse (Table 2). For glioma CLXs, immortalized glioma cell lines commonly used for implantation include U87, U251, T98G and A172 [22,23]. CLXs are a quick and reproducible strategy for studying glioblastoma. However, they often result in well-circumscribed tumors that lack the characteristic infiltrative pattern that is observed in human gliomas [22,23]. Further, the selective pressures of cell culture reduce the sub-clonal heterogeneity of CLXs and their ability to recapitulate the parent tumor [24]. Advances in CLX modeling have been driven by the isolation and propagation of glioma stem-like cell (GSC) lines, which are commonly identified by the cell surface antigen CD133 and retain expression of stemness markers that are readily observed in human glioma [25]. GSC lines are propagated as neuro-sphere cultures using ultra-low adherence culture vessels and serum free media containing neural cell-specific growth factors. In contrast to CLX models, patient-derived xenografts models (PDX) involve direct xenotransplantation of human biopsy tissue (Table 3). Importantly, PDX models are exposed minimally, if at all, to in vitro culture, which avoids adaptation to non-physiological conditions and preserves features of the tumor of origin. PDX models have been shown to better recapitulate the vascular characteristics and blood brain barrier permeability of patient HGGs as compared to CLX tumors derived from the U87 cell line [22,23]. Thus, PDX models are better suited to recapitulate the stromal and interactions and invasiveness of parent tumors than their CLX counterparts. Xenograft models have been used extensively in the study of glioma biology [26,27,28]. In 1986, Kaye et al. were one of the first to create a model using this system by implanting a C6 glioma cell line (a rat glioma cell line) into neonatal and adult mice to demonstrate a reliable murine xenograft glioma model [27]. When creating a reliable xenograft model, the location of cancer cell implantation needs to be precise for development of a tumor that accurately recapitulates the human counterpart [28]. Iretenkauf et al. (2017) utilized a glioma xenograft model with nude mice and showed that the implantation location of GSCs within the brain can affect the developed tumor characteristics in the murine model [28].

### Immunology Research in Xenograft Models

Xenograft models have several benefits including low-cost and fast throughput [122]. A limitation of xenograft models is the required use of immune-deficient mice.

Immune-deficient mice used in xenograft models include nude mice, non-obese/diabetic mice (NOD), severe combined immunodeficient mice (SCID), and the combination NOD/SCID and NOD/SCID/interleukin IL2 receptor γ_null_ (NSG) mice. The nude (athymic) mouse has a depleted population of T lymphocytes acquired through mutations in FOXP1 [123]. Nude mice have increased NK cell and macrophage activity as well as intact B cells, dendritic cells, and granulocytes [124]. Thus, while unable to characterize the lymphocyte mediated response, nude mice models can provide information on other immune cell interactions with the tumor [124]. Another common mouse utilized is the SCID mouse which lacks mature B and T lymphocytes [125]. NSG mice carry significant reductions in natural killer cell function in addition to B and T lymphocyte loss to reduce the innate and adaptive immune system for successful grafting of more immunogenic tumors [126]. These immune-compromised mice are necessary for the successful engraftment of tumors without risk of short-term rejection. Loss of the immune microenvironment limits study of tumor interaction with the immune system and testing of immune modulating agents [127,128]. Recent studies show that humanized mouse models may help to overcome this challenge [127].

Humanized mouse models are used to generate a mouse with a competent human immune system to study immune responses to anti-cancer immunotherapies [129]. They are created with NSG or NOD/SCID mice undergoing whole body irradiation followed by injection of human CD34+ hematopoietic stem cells intravascularly [124,130]. After 12 weeks of age, successful engraftment of the human immune system can be assessed with flow cytometry [129]. These humanized mice are then injected with patient derived tumor tissue to develop into humanized PDX models [129].

An alternate method to study immune systems in PDX models was proposed by Semenkow et al. (2017), who demonstrated that blocking T-cell co-activating signals with immune checkpoint inhibitors, abatacept and MR1, allowed for short term tumor development in orthotopic glioma murine models with intact immune systems [131]. Both models are cost- and time-intensive but add to the current and future understanding of immune modulation on tumorigenesis and progression.

## 4. Syngeneic Models

Syngeneic glioma rodent models have been generated via injection of the carcinogen ethyl nitrosourea into the placenta between the 15th and 18th day of murine pregnancy [22]. Additional syngeneic models, namely GL261 and CT-2A, were produced by intracranial injection of the carcinogen 3-methylcholantrene, leading to formation of tumors that resembled GBMs. Cell lines derived from these brain tumors can be used to create syngeneic allografts upon transplantation into naïve mice from the same genetic background [22,128,132]. Unlike xenograft models, syngeneic models utilize immune-competent animals. This allows the study of the interaction between the tumor and immune microenvironment, and the possibility of testing immunotherapies for cancer treatment. Like other models that are based on cell line propagation, the syngeneic mouse model is subject to genetic drift with long term propagation [22]. In addition, given that syngeneic models exclusively involve mouse tissue, they present challenges for translating findings to human cancer. Gliomas induced in mice through carcinogen exposure present as well-circumscribed tumors without infiltration into the surrounding brain parenchyma, which is not the typical growth pattern appreciated in human astrocytoma [22]. Therefore, these models do not fully recapitulate the morphological characteristics of human glioma [14,133].

## 5. Genetically Engineered Mouse Models (GEMMs)

GEMMs involve manipulation of the mouse genome to induce tumor formation [124]. By causing autochthonous tumors to form in mouse tissue, immune-competent mice can be utilized, a key advantage over xenograft models. The intact immune system and native tumor cytoarchitecture enable studies of the tumor microenvironment, while genetic engineering affords precise control over the molecular events leading to tumor formation, maintenance, and susceptibility to treatment [124]. Furthermore, GEMMs allow for the ability to activate relevant oncogenes at specific time points in tissue development, and they permit testing of potential therapeutic agents at various stages of tumorigenesis. These properties offer distinct advantages over PDX models, which are nearly universally derived from advanced human tumors.

GEMMs are frequently made with inbred mouse strains similar to those used in syngeneic models. The C57BL/6 mouse strain, established in the 1920s to study immune responses to cancer, has an increased NK cell activity and high cell-mediated immune response, but a weak antibody-mediated response [134]. By comparison, BALB/c mice have a stronger humoral immune reaction [135]. FVB/N mice (also known as friend virus B-type susceptibility), were created in the late 1970s from the Swiss N:GP mice (also known as the National Institute of Health general purpose mouse) [136]. In relation to BALB/c mice, FVB/N mice have been shown to respond with a greater Th2 bias; however, the immune status is poorly defined [137].

Historically, challenges with timing, sufficient tumor development, and inability to recapitulate the intra-tumoral heterogeneity of gliomas made it difficult to utilize GEMMs for in vivo glioma modeling [124]. Advancements in these GEMMs have created several modeling systems that better recapitulate human gliomas. These include development of the replication competent avian-like sarcoma virus and the corresponding avian tumor virus A (RCAS-tVA) system, the Cre-loxP system, and the sleeping beauty transposon system (Table 4).

### 5.1. Somatic Gene Delivery Models

The RCAS-tVA system allows for oncogenes to be transferred to cells that express the tVA receptor using a cell type-specific promoter [22]. Importantly, the avian virus used in this system does not replicate in mammalian cells. Therefore, the interaction between induced tumor cells and healthy cells remains intact and can be evaluated without the potentially confounding effects of viral propagation [22,159]. Genetic mutations arising in single cells and cells selectively undergoing clonal expansion can be demonstrated by this model [160]. Tissue-specific promoters can be used to selectively introduce oncogenes into nestin-positive neural stem and progenitor cells (Ntv-a), glial fibrillary acidic protein (GFAP) -positive glial cells (Gtv-a), or CNPase-positive oligo-dendroglial cells (Ctv-a), thereby enabling studies of the tumor cell of origin in glioma GEMMs [159].

Holland and Varmus (1997) were the first to use an RCAS-tVA transgenic mouse model to demonstrate induction, proliferation, and migration of glial cells with β-FGF [159,160,161]. They also subsequently showed that EGFR mutations in murine glial cells induce lesions that are similar to human gliomas [162]. EGFR-induced gliomas also form in transgenic mice with a INK4a-ARF tumor suppressor locus disruption [162].

While the RCAS-tVA system is limited by the vector capacity of the RCAS virus, other viruses have been used for somatic gene transfer and GEMM production, including adeno-associated viruses (AAVs), adenoviruses, and lentiviruses [22,160]. Marumoto et al. (2009) successfully utilized a Cre-loxP controlled lentiviral vector expressing activated Harvey-Ras and AKT to recapitulate simultaneous activation of oncogenes in a few cells to initiate the formation of grade III and IV gliomas in immune-competent adult mice [158]. The advantage of these viruses in comparison to the RCAS virus is the ability to infect both dividing and non-dividing cells [159].

### 5.2. Conditional Allele-Specific Models

The Cre-loxP system utilizes the Cre recombinase enzyme to induce recombination between two loxP recognition sites [22]. Conditional models involve breeding a tissue-specific transgenic Cre recombinase mouse with a mouse in which a gene (or genes) of interest has been flanked with loxP sites through a knock-in approach [163]. An inducible Cre-loxP system is created by placing Cre protein activity or gene expression under control of tamoxifen (Cre-ER) or tetracycline (Tet-On/Off) [164]. Cre-loxP systems offer highly efficient genetic modification and have been utilized to create mice that develop GBMs through the introduction of EGFRvIII mutations [22,163]. Cre recombinase expression cassettes can be placed under the control of brain-specific promoters, such as nestin or GFAP, to achieve cell type-specific genetic engineering [165,166].

Cre-loxP systems have been utilized to evaluate the relationship between NF1 and glioma formation. C57BL/6 mice with NF1 mutations inbred with C57BL/6 mice with TP53 mutations developed malignant glial neoplasms of the central and peripheral nervous system [167]. Zhu et al. (2005) demonstrated that mice with NF1 and p53 mutations develop WHO grade II gliomas that progress to anaplastic astrocytoma and GBMs [140,167].

### 5.3. Transposon/Transposase Models

The sleeping beauty system can be used to identify genetic drivers in animal models in an unbiased manner [22]. This system is thus important in understanding glioma-genesis [22]. Bender et al. (2010) utilized a T2/onc transposon with a constitutively active sleeping beauty transposase to create a high grade astrocytoma. The resulting gliomas displayed an invasive phenotype and expressed GFAP and S100 markers, which are characteristic of human brain tumors, indicating that this is an effective system to model glioma formation [157].

## 6. Special Consideration for IDH1/2 Mutations

Overall, approximately 80% of lower-grade gliomas and secondary glioblastomas harbor an IDH1 or IDH2 mutation, thus supporting the inclusion of this class of mutations in mouse models of these glioma subtypes [155]. The IDH1-R132H mutation is by far the most common IDH1 or IDH2 mutation observed in glioma. IDH1/2 mutant oncoproteins harbor point mutations in arginine residues that line these enzymes’ active sites. Wild-type IDH enzymes normally convert isocitrate to alpha-ketoglutarate (α-KG), whereas IDH mutant oncoproteins gain the neo-morphic ability to convert α-KG into D-2-hydroxyglutarate (D-2-HG), a compound normally found in small intracellular quantities [168]. High levels of D-2-HG competitively inhibit α-KG-dependent enzymes, leading to widespread epigenetic and metabolic reprogramming [169]. Importantly, although low grade gliomas with IDH mutations carry a relatively favorable prognosis, these tumors inexorably progress to high grade glioma and are universally fatal. Thus, creating accurate mouse models of IDH mutant glioma to study the unique pathobiology of these tumors is imperative.

Philip et al. (2018) utilized an RCAS-Ntv-a system to create an IDH1 mutant glioma model by expressing the IDH1-R132H oncoprotein and platelet derived growth factor receptor A (PDGFRA), and simultaneously silencing CDKN2A, alpha thalassemia/mental retardation syndrome x-linked (ATRX), and PTEN [170]. Heterotopic and orthotopic IDH1 mutant glioma xenografts are also utilized to model this disease state [22]. Borodovsky et al. (2015) utilized fresh patient tissue to create a subcutaneous IDH1 mutant tumor that was serially propagated [168]. Later, dissociated cells were implanted into nude mice orthotopically and displayed IDH1 mutant anaplastic astrocytoma formation leading to the creation of the JHH-273 murine model [168]. Orthotopic xenograft models of IDH1 mutant GBM, including the MGG152 model, have also been used to identify new therapeutic targets in this disease, including the discovery of NAD+ biosynthesis as a novel metabolic vulnerability conferred by IDH oncogenes [171].

Establishing new models of other IDH mutant glioma subtypes is also important for pre-clinical testing of new therapeutic strategies. The TS603 subcutaneous xenograft model of oligodendroglioma (featuring pathognomonic codeletion of 1p/19q chromosome arms and mutation of IDH1) has been utilized to test the antitumor efficacy of the mutant IDH1 inhibitor AGI-5198 [172]. Importantly, AGI-5198 blocked growth of TS603 xenografts, thus paving the way for clinical trials of mutant IDH inhibitors in glioma [172]. Schumacher et al. (2014) utilized a humanized murine model to demonstrate that a mutant IDH-specific vaccine induces interferon gamma-producing T-cells that target IDH1 mutant tumor cells [173,174]. There are currently on-going clinical trials to assess if these therapeutic approaches will be effective treatment strategies for patients with low grade gliomas [174].

Much like IDH1/2 mutations, improved understanding of epigenetic dysregulation in glioma has led to the discovery of mutations encoding a lysine to methionine substitution at position 27 in histone H3 (H3K27M), which leads to the formation of high-grade gliomas, especially diffuse midline gliomas in children [175,176,177]. The introduction of newly identified mutations into murine models of glioma is expected to open new avenues for preclinical investigation of novel glioma onco-genotypes in future studies.

## 7. Conclusions

Glioma animal models offer substantive advantages over in vitro two-dimensional glioma cell cultures as they better recapitulate the genetic, morphologic, and immunologic characteristics of human tumors. Since the development of the first murine cancer xenograft model 70 years ago, there have been many advances, including the creation of PDXs and GEMMs. These developments have allowed for the creation of faithful glioma models to study the genetic and molecular changes driving glioma-genesis, immunologic tumor recognition, and therapeutic response. The development of innovative new glioma murine models provides opportunities to study the process of glioma-genesis in greater detail and to evaluate the safety and efficacy of experimental treatments more accurately in the preclinical setting.

## Figures and Tables

**Table 1:** Comparison of preclinical animal model features.

Model	Tumor Source	Immune Status	Cost	Labor/Time
CLX	Human	(−)	$	+
PDX	Human	(−)	$$	++
Syngeneic	Mouse	(+)	$	++
GEMM	Mouse	(+)	$$$	+++

CLX, cell-line xenograft; PDX, patient-derived xenograft; GEMM, genetically engineered mouse model.

**Table 2:** Glioma cell-line xenograft (CLX) and syngeneic murine models.

Mouse Species	Brain Tumor Modeled	Tumor Cell Line	Reference
BALB/c OlaHsd-Foxn1^nu^	Glioma	BT3 cells	[29]
C57BL6/N	High Grade Glioma	GL261 cells	[30,31,32,33,34,35,36,37]
	High Grade Glioma	U87 and GL261 cells	[38]
CBA, BALB/c, AKR, C57 black, and RIII	Glioma	Rat C6 cells	[27]
CD-1, Nude, and NOD CRISPR Prkdc IL2Rγ_null_	Glioma	DAOY and T98G cells	[39]
CIEA-NOG	Glioblastoma (GBM)	Patient derived glioma cell lines	[40]
C6B3F1	High Grade Glioma	Mouse Tu2449, Tu9648 and Tu251 mouse glioma cell lines	[41]
Foxn1^nu/nu^	Glioma	BT4C cells	[29,42]
ICR	High Grade Glioma	C6 rat glioma cells	[43]
Non-obese/diabetic (NOD)/Severe combined immunodeficient (SCID)	Isocitrate dehydrogenase 1 (IDH1) Mutated Glioma	Patient derived IDH1 mutant oligoastrocytoma	[44]
	GBM	TG1 human GBM cell line	[45]
	GBM	T98 and U87 glioma cell lines	[46]
Nude/NOD/SCID	High Grade Glioma	U87, U118, LN18, LN229 cell lines	[47]
Not reported	GBM	U87 and U373 glioma cell lines	[48]
Nude	GBM	Ink4a/ARF^−/−^ Id4 astrocyte cells	[49]
	Malignant Astrocytoma	Commercial malignant cell lines	[50]
	High Grade Glioma	BT70 malignant glioma cell line	[51]
	High Grade Glioma	U87 human glioma cell line and C6 rat glioma cell line	[52]
	GBM	LN229 and U87 human glioma cells	[53]
	High Grade Glioma	E98 and E473 glioma cell lines	[54]
	GBM	Mouse GL261 cell line	[55]
	GBM	Human U87 glioma and rat 9L glio-sarcoma cell lines	[56]
	GBM	Patient derived GBM cell lines	[57,58,59]
	High Grade Glioma	Human astroglioma U373 and T98G and oligodendroglioma Hs683 cell lines	[60]
	High Grade Glioma	Human glioma U87, U251, U373, A172, LN18, LN229, and D54 cell lines	[61]
	High Grade Glioma	Hs683 cells	[62]
	High Grade Glioma	LN229 cells	[63,64]
	High Grade Glioma	SHG44 cells	[65]
	High Grade Glioma	T98G and U373 cells	[66]
	High Grade Glioma	U87, U251 and D566 cells	[67]
	High Grade Glioma	U87 cells	[68,69,70,71,72,73,74,75,76,77,78,79,80,81,82,83,84,85,86,87,88,89,90]
	GBM	U87. LNZ308, LN229 cells	[91]
	High Grade Glioma	U87, U118, N10, U251, A172, and U373 cell lines	[92]
	High Grade Glioma	U251 cell line	[93,94]
	GBM	U87 and LN229 cell lines	[95]
	GBM	LN229 cell line	[96]
	Glioma	E102 and E106 glioma cell lines	[97]
	Glioma	SNB-19 U87 glioma cell lines with co-transfecting COS-7 cells with pTet-On and treated with doxycycline	[98]
	High Grade Glioma	Human T269 4IgB7H3 knockdown or control cells (orthotopic); LN-229 4IgB7H3 knockdown or control cells (subcutaneous)	[99]
	High Grade Glioma	U87 and U251 glioma cell lines	[100]
	Glioma	A-172, U343, U87 and T98G glioma cells	[101]
	Glioma	U87 glioma cell line	[102]
	Glioma	U373 human glioma cell line	[103]
SCID	High Grade Glioma	GLI36-EGFRvIII engineered cells	[104]
	Glioma	Patient-derived GSC lines	[105,106,107]

**Table 3:** Glioma patient-derived xenograft (PDX) murine models.

Mouse Species	Brain Tumor Modeled	Source of Tumor Cells	Reference
eGFP NOD/SCID mice	Oligodendroglioma	Patient-derived tumor cells	[108]
NOD-Prkdc^SCID^ IL2Rγ_null_	Grade II-IV Glioma	Patient-derived glioma tissue	[109]
NOD-SCID	Malignant Astrocytoma	Embryonic stem cells	[110]
	Glioma	Patient-derived high grade glioma tissue	[111]
NOD/SCID Il2rg^−/−^ (NOG)	GBM	Patient-derived GBM cells	[112]
Not reported	GBM	Patient-derived human GBM cells	[113]
NSG	GBM	Patient-derived GBM neuro-spheres	[114]
Nude	Glioma	Patient-derived IDH mutant glioma tissue	[115]
	GBM	Patient-derived GBM tissue	[116,117]
	GBM	Patient-derived GBM tissue	[28,118]
SCID	IDH Mutant Glioma	Patient-derived glioma neuro-spheres	[119]
	IDH1 Mutated Glioma	GBM164, GBM196, and TB09 IDH1 mutant glioma PDX models	[120,121]

**Table 4:** Glioma genetically engineered mouse models (GEMMs).

Mouse Species	Brain Tumor Modeled	Genetic Engineering	Reference
C57BL/6 and Tp53^−/−^	GBM	PDGFβ, p53 mutations	[138]
C57BL/6	Glioma	Heterozygous TgGZT_121,_ KRAS^G12D^, GFAP-CreER, PP-CreER, NG2-CreER, and Rosa26-tdTomato mice crossed with mice with conditional PTEN, p53, Rb1, or NF1 loss	[139]
	Glioma	Crossing of NF1^flox+^ mice with p53^+/−^ mice and then crossed with wild type F1 C57BL/6 mice	[140]
	High Grade Astrocytoma	RB, phosphate and tensin homolog deleted on chromosome 10 (PTEN) mutations	[141,142]
Crossed IDH1 and Nestin-Cre transgenic mice	IDH1 R132 Mutated Glioma	Nestin-Cre remodeling system	[143]
FVB/N mice	Oligodendrocyte	Ctv-a plasmid was transfected into an immortalized oligodendroglia cell line OLI-neu	[144]
FVB/N, C57BL/6, BALB/C, and 129	GBM	K-Ras, Akt, Ink4a/Arf mutations	[145,146]
	GBM	Platelet derived growh factor receptor (PDGF)β, Ink4a/Arf, PTEN mutations	[147]
Gtv-a Arf^−/−^	High Grade Glioma	Induction with RCAS-PDGF-B	[148]
INK4a^+/+^ and INK4a^−/−^	GBM	PDGFβ	[149]
	IDH1 mutant and wildtype gliomas	PDGF, Tp53, and IDH1 mutations	[150]
MUT3 (Mice with mixed genetic background of C57BL/6, Sv129 and B6/CBA)	De novo GBM	Introduced PTEN and p53 flexed alleles into MUT3 mice	[151]
Not Reported	High Grade Glioma	K-Ras, p53, Ink4a/Arf mutations	[152]
	GBM	EGFRvIII, Ink4a/Arf, PTEN mutations	[153]
	GBM	PDGFβ mutation	[154]
	Lower- and Higher-Grade Gliomas	Neuroblastoma RAS (NRAS) G12V and shp53 Sleeping beauty plasmids with or without shATRX and IDH1^R132H^	[155]
RasB8	High Grade Glioma	EGFRvIII and V^12^ Ras mutation	[156]
Rosa26-SB11	High Grade Glioma	T2/onc mutagenic transposon	[157]
GFAP-Cre transgenic mice maintained with C57BL/6 and crossed with Tp53^−/−^ mice	High Grade Glioma	H-Ras, AKT and Tp53 mutations	[158]

## Data Availability

Not applicable.
